# The environmental threats from lead ammunition

**DOI:** 10.1016/j.eehl.2023.02.001

**Published:** 2023-02-14

**Authors:** Christian Sonne, Su Shiung Lam, Niels Kanstrup

**Affiliations:** a Aarhus University, Faculty of Technological Sciences, Department of Ecoscience, DK-4000 Roskilde, Denmark; b Sustainability Cluster, School of Engineering, University of Petroleum & Energy Studies, Dehradun, Uttarakhand 248007, India; c Higher Institution Centre of Excellence (HICoE), Institute of Tropical Aquaculture and Fisheries (AKUATROP), Universiti Malaysia Terengganu, 21030 Kuala Nerus, Terengganu, Malaysia; d University Centre for Research and Development, Department of Chemistry, Chandigarh University, Gharuan, Mohali, Punjab, India; e Department of Ecoscience, Aarhus University, C.F. Møllers Allé 8, 8000 Aarhus C, Denmark

## Abstract

• Lead (Pb) is an extremely neurotoxic persistent element in the environment;
• Negative environmental impact continues due to the use of lead ammunition;
• Lead ammunition contributes 93,000 tons of lead to the EU environment alone;
• Environmental losses from lead account for 1 billion EUR/year in Europe alone;
• A One Health approach may lower the environmental threats from lead.

## Graphical abstract

**Figure:**
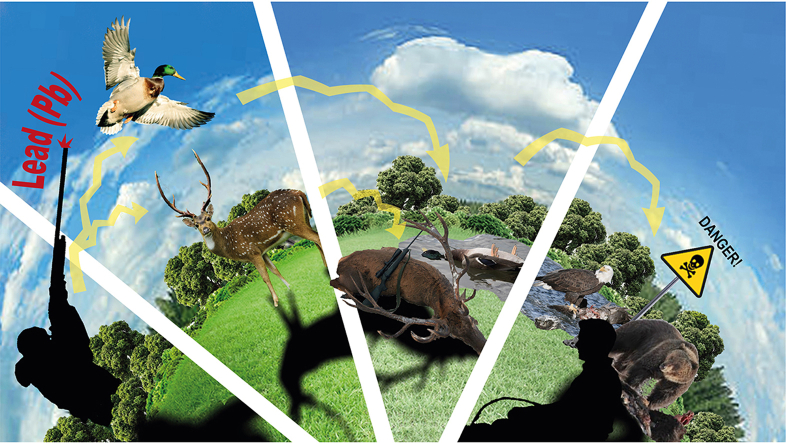

Lead (Pb) is an extremely toxic persistent element, considered a factor in the fall of the Roman Empire 2000 years ago due to its use in plumbing and as a wine sweetener [1]. The effects of lead include adverse neurological effects, reproductive impairment, and anaemia [2]. One continuing source of lead in the environment is lead ammunition, which presents health risks to consumers of game meat, including humans, predatory and scavenging birds, and other animals. In wild birds, ingestion of lead from ammunition can cause sub-lethal effects, death, and affect population levels [[2], [3], [4], [5]]. A new study shows that the dispersal of lead ammunition from rifle bullets and shotgun pellets costs 1 billion EUR/year from losses in wildlife, environmental status, and socio-economy, just in Europe [[6], [7], [8]]. The European Chemicals Agency (ECHA) estimates for EU-27 that, from hunting, 5000–7000 tonnes of gunshot lead are deposited into wetlands annually, and that an additional 13,000–15,000 tonnes are released into other habitats giving a total of 18,000–22,000 tonnes a year [9]. On top of that, 35,000 tonnes of lead from gunshot and 42,000 tonnes from lead bullets are released from sport shooting activities, although this has a more restricted distribution compared to the release from hunting, which is distributed widely into natural habitats. There is no lower tolerable lead intake for humans, according to the European Food Safety Authority (EFSA), reflecting the need to increase the awareness of health risks from lead-contaminated game meat [5,10].

Secondary lead poisoning from the use of lead ammunition has been shown to cause suppression of raptor populations worldwide [11,12]. Recently, the Danish EPA announced a ban on all types of lead ammunition for hunting, which is the first country to do so [8]. This will take effect from April 2024 and is inspired by Denmark’s previous ban on lead gunshot pellets leading to a significant decline in game meat content over the past two decades [13], alleviating risks to ecosystems and human health. While lead concentrations in game meat have decreased to very low levels in Denmark, they have increased elsewhere in Europe over the last decade. These countries typically have no or only partial restrictions on lead ammunition, and demonstrate lead in game meat being fourteen times higher than the levels used in EU-wide risk assessments [13]. Following on from Denmark, the EU has introduced a phase-out of lead gunshot pellets in wetlands by 2023 mainly to prevent primary poisoning of water birds from ingestion of spent lead shot (Regulation (EU) 2021/57). In addition, the EU has proposed a restriction on the use of lead ammunition in sport shooting and all other types of habitats for hunting, with a decision expected in 2023 [8,[14], [15], [16]]. Unfortunately, this is far from sufficient and the magnitude of the problem connected to lead ammunition calls for a complete ban on all lead hunting and sports ammunition worldwide.

Nearly half of the golden eagle (*Aquila chrysaetos*) and bald eagle (*Haliaeetus leucocephalus*) populations in the United States carry lead concentrations that suppress population growth [11]. Other raptors, such as the California (*Gymnogyps californianus*) and Andean (*Vultur gryphus*) condors, are classified by IUCN (The International Union for Conservation of Nature) as Critically Endangered and Vulnerable, respectively, lead being a major factor in the recent near-extinction of the California condor [[17], [18], [19]]. These issues are not isolated to North and South America but are a problem for raptors across continents. New analysis of Swedish golden eagles and white-tailed eagles (*Haliaeetus albicilla*) shows how lead intoxication from spent hunting ammunition has increased over the past decades [20]. Similar findings include wedge-tailed eagles (*Aquila audax*) in Australia and Steller’s sea eagles (*Haliaeetus pelagicus*) in Japan as well as African vultures [4,7,21,22].

Safe and effective alternatives to lead ammunition are available on the market or, when locally absent, will be so once the demand for such products is ensured through the phase-out of lead ammunition through effective regulation. This applies to non-lead gunshot as well as rifle bullets [23,24].

The continued use of lead ammunition threatens biodiversity and human health, impedes sustainability and challenges the One Health approach [25]. We therefore urge international and national authorities across all management sectors, together with relevant citizens and stakeholders, to collaborate and encourage hunters and the ammunition industry to phase out the use and production of lead-hunting ammunition and to transit to non-toxic alternatives as soon as possible. A One Health approach would benefit the interests of hunters by reducing losses of quarry animals through poisoning and stimulating a more positive public perception of hunting. These actions are much needed given the current situation where ammunition lead deposited in natural habitats or embedded in hunted animals constitutes an unstainable and avoidable pollution challenge that must and can be solved [6]. Only such holistic use of a worldwide One Health approach may lower the current ecosystem and environmental threats from lead on humans, wildlife, and ecosystems [5,8,26].

## Declaration of competing interest

The authors declare no conflicts of interest.
